# Using Benthic Macroinvertebrate and Fish Communities as Bioindicators of the Tanshui River Basin Around the Greater Taipei Area — Multivariate Analysis of Spatial Variation Related to Levels of Water Pollution

**DOI:** 10.3390/ijerph110707116

**Published:** 2014-07-14

**Authors:** Shuh-Sen Young, Hsi-Nan Yang, Da-Ji Huang, Su-Miao Liu, Yueh-Han Huang, Chung-Ting Chiang, Jin-Wei Liu

**Affiliations:** 1 Department of Applied Science, National Hsinchu University of Education, 521 Nan-Da Rd. Hsinchu, 300, Taiwan; E-Mails: maoluffy@hotmail.com (Y.-H.H.); chargy0127@gmail.com (C.-T.C.); sdfvgsdfvg@gmail.com (J.-W.L.); 2 Environmental Analysis Laboratory, Environmental Protection Administration, Executive Yuan, 260 Minzu Rd. Section 3, Zhongli City Taoyuan County 32024, Taiwan; E-Mails: hnyang@mail.niea.gov.tw (H.-N.Y.); smliu@ mail.niea.gov.tw (S.-M.L.); 3 Department of Environmental Resource Management, Chia-Nan University of Pharmacy and Science, 60 Er-Ren Rd. Section 2, Ren-De Division, 71710 Tainan City, Taiwan; E-Mail: daji@ms19.hinet.net (D.-J. H.)

**Keywords:** bioindicator, benthic invertebrate, fish community, water quality

## Abstract

After decades of strict pollution control and municipal sewage treatment, the water quality of the Tanshui River increased significantly after pollution mitigation as indicated by the River Pollution Index (RPI). The pollution level of the estuarine region decreased from severe pollution to mostly moderately impaired. The most polluted waters are presently restricted to a flow track length between 15–35 km relative to the river mouth. From July 2011 to September 2012, four surveys of fish and benthic macroinvertebrates were conducted at 45 sampling sites around the Tanshui River basin. The pollution level of all the study area indicated by the RPI could also be explained by the Family Biotic Index (FBI) and Biotic Index (BI) from the benthic macroinvertebrate community, and the Index of Biotic Integrity (IBI) of the fish community. The result of canonical correlation analysis between aquatic environmental factors and community structure indicated that the community structure was closely related to the level of water pollution. Fish species richness in the estuarine area has increased significantly in recent years. Some catadromous fish and crustaceans could cross the moderate polluted water into the upstream freshwater, and have re-colonized their populations. The benthic macroinvertebrate community relying on the benthic substrate of the estuarine region is still very poor, and the water layer was still moderately polluted.

## 1. Introduction

**Figure 1:**
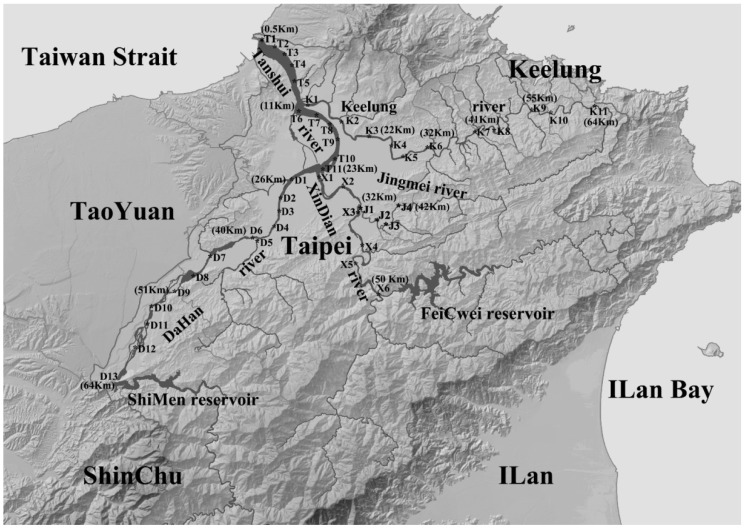
Tanshui River system and sampling site on each branch. T1-T11 for Tanshui River, K1-K11 for Keelung River, X1-X6 for Xindian River, J1-J4 for Jingmei River and D1-D13 for Dahan River. The distance to the sea of the flow track for each sampling site is indicate in parentheses (km).

The Tanshui River is fed by two major branches at Hua-Jiang bridge, the Xindian River and Dahan River; near the river mouth, another branch, Keelung River, converges at Gwndwu. Including the Jingmei River (a branch of the Xindian River), the lower part of Tanshui River basin flows through all the Greater Taipei urban region, which is inhabited by 7 million people. The Keelung River, Xindian River and Dahan River have been the key freshwater sources for city development since time immemorial. In 1970s, the lower part of Tanshui River was seriously impaired because of quick urbanization, industrialization, and human population growth [1,2,3,4,5]. The most deleterious pollution sources were upstream coal mining and sewage discharge by the aggregation of villages along the riverside. The coal mining sites were scattered in the mountain range around the Greater Taipei basin. Along the Keelung River, the mining distribution ranged from Rwei-Fang to Xi-Zhi (sites K7-K10 in Figure 1). San-Xia township to near the Dahan River (site D5 in Figure 1), and there was a major mining site at Hai-Shan. Mining also could be found at the Jingmei and Xindian area, and the Shih-Dieng mining site (Jingmei River; site J4 in Figure 1) and An-Keng mining site (Xindian River; site X3 in Figure 1) were two spots near the river. The scale of mining decreased after 1984, because of a serious mining disaster that killed hundreds of people. After the 1980s, water pollution was not eased and became more severe at the 1990s when the human population increased dramatically. Before 1990, river management mostly focused on the construction of protective embankments and took advantage of the riverbed above the high water line as publicly-owned land. Public land on the riverbanks was used as football fields, basketball fields, roads for cars and bikes, parking lots, etc. Between 1990 and 2000, truncation curved cut-off and canalization physically altered the Keelung River.

Specific water pollution regulation and sewage treatments were underway after 1990. The BaLee sewage treatment plant entered service in 1998. The Neihu sewage treatment plant has functioned since 2004. The Dihua primary treatment plant built in 1970, was upgraded to secondary treatment ability in 2006. The Neihu sewage treatment plant improved its denitrification ability in 2008. On site wetland primary treatment facilities were constructed on the riverbanks of the Xindian River and Dahan River from 2000 to 2010. According to the report on website of the Construction and Planning Agency, Minister of Interior, the sewage water treatment rate of Taipei City was near 100% in 2010 [6].

The pollution mitigation of the Tanshui River was achieved by sewage treatment in recent years. According to the report on the website of the Taiwan Environmental Protection Agency, the water quality of Tanshui River is classified as lightly to moderately polluted in most area, rather than seriously polluted as at the beginning of 2010 [7]. The river pollution index (RPI) is a composite indicator of the chemical and physical properties of water, which is used by the Taiwanese Environmental Protection Administration (EPA) to evaluate the water quality (Table A1). The water quality of Tanshui River in 2012 was much better than in 1990s as indicated by the RPI. The ecological improvement was still not understood by ecologists. Water quality or RPI could respond to specific wastewater treatment activities more quickly than the biological community. Aquatic organisms have different life stages with different habitat requirements, so short term aquatic environment improvements might not ensure their survival. The biotic community structure can be a more adequate indicator to interpret the long term effects of water quality improvement.

For livelihood water supplies, micro-organism content (total viable bacterial count (TVBC), coli-form group, mold and yeast count, *Enterococcus* and sulfur-oxidizing bacteria) are common items used to evaluate water quality. Including the micro-organisms, the assemblage of macrophytes, microalgae, invertebrates and fish has been used to monitor water quality in different aquatic ecosystems for different purposes in recent years [8,9,10,11,12,13,14,15,16,17,18,19]. The earliest biotic index of a saprobic system was developed about 100 years ago [20,21,22], using invertebrates as indicators.

Biological indicators have long been used in ecological assessments of surface water quality by the U.S. EPA after the passage of the Federal Water Pollution Control Act Amendments of 1972. In 1991, the U.S. EPA issued a policy statement regarding “Use of Biological Assessments and Criteria in the Water Quality Program” [23]. In recent years, biological assessments by the U.S. EPA have emphasized more the biological integrity of aquatic systems rather than simple indicators [24]. The Water Framework Directive (WFD) of the European Union developed a series of practical assessment tools for water managers in 2006. For river eutrophication, macrophytes, phytoplankton and phytobenthos were the key biological groups to assess the impact of inorganic nutrient enrichment in river ecosystems [25]. For organic pollution in rivers, the WFD required the use of five biological elements to assess the impacts: macrophytes, phytoplankton, benthic algae, benthic invertebrates and fish. Among these five key biological elements, benthic algae and invertebrates were widely used as indicators in the EU. The assessment of water quality using bioindicators, in a first approach considered multimetric indices to incorporate into a single value different components of the biological community. The second approach was using multivariate statistical methods to uncover the patterns in taxonomical composition related to environmental variables [26].

The algae community (phytoplankton and phytobenthos) structure is commonly used to evaluate the eutrophication and organic pollution of river and lakes in Taiwan. The most commonly used index was the Genera Index (GI) created by Wu [27]. The benthic invertebrate and fish assemblage are also in use to assess the pollution level of rivers. The Hissenhoff Family Biotic Index (FBI) [28] and Biotic Index (BI) [29] derived from benthic invertebrates are used as water quality indicators more frequently. The Index of Biotic Integrity (IBI) derived from fish assemblages and created by Karr [30] is also useful to study the effects of pollution on fish communities. Microorganisms and algae have short life cycles, so their community structure response to water quality in the short term could be a good indicator after short term pollution disturbances. Benthic invertebrates and fish with longer life cycles may more easily suffer from the water pollution that is prolonged for a long period. In the perspective of long term effects of pollution mitigation, we used the benthic invertebrate and fish community as the bio-indicators to evaluate the spatial variation of water quality of the Tanshui River basin in 2012. Multivariate statistical methods were employed to uncover the patterns in taxonomical composition related to the levels of water pollution.

## 2. Experimental Section

### 2.1. Basic Environmental Variables

From July 2011 to September 2012, four surveys (October 2011; January, April and July 2012) were conducted around the Tanshui River basin. We had 45 sampling sites in total—Xindian River with 10 sites (including four sites along the Jingmei River branch), Dahan River with 13 sites, Keelung River 11 sites, and Tanshui River with 11 sites (Figure 1).

The samplings of the lower region affected by tidal movement were conducted on a small boat during flood tide. Onsite measurement of water properties included temperature (°C), conductivity (μs/cm), salinity (PSU), dissolved oxygen (mg/L), and pH scale by HORIBA U-50 and HACH *sension* 156 Multiparameter Meter portable instruments. Conductivity, temperature, and depth profile (CTD: SBE 19 plus SEACT PROFILER; SBE 18 pH sensor; SBE 43 Sea Bird Dissolved Oxygen Sensor) also used in the downstream areas with depths of more than 1 meter. In each sampling, water samples were collected to verify the biochemical oxygen demand (BOD_5_ mg/L: standard method NIEA W510.55B), ammonia nitrogen (mg NH_3_—N/L: standard method NIEA W446.52C), and suspended solids (SS mg/L: standard method NIEA W210.57A). River pollution index (RPI) was composed by DO, SS, BOD_5_ and ammonia nitrogen according to EPA Taiwan (Table A1).

### 2.2. Biological Sampling

Different collection methods were used for sampling of benthic invertebrates depending on the river substrate. On the upstream river bed, we used a Surber net to collect the epifauna living on the rocks and coarse sand in shallow waters. Each sampling site collected 3–5 subsamples, each subsample covering 900 cm^2^. On the downstream river bed, we employed a Ponar dredge to collect 5–6 subsamples of epi-fauna and in-fauna living in the soft substrate under deep water. The grabbed substrate was screened by a No. 35 (500 μm) sieve to trap the benthic organisms. Each Ponar dredge subsample covered 17 × 25 cm^2^. All the raw materials sampled in the wild were stored in a jar and preserved under low temperature onsite. Samples collected in the wild were transferred to laboratory frozen below −20 °C. The frozen material was thawed at room temperature, the benthic organisms sorted under a stereo-microscope and the specimens preserved in 70% alcohol. The specimens were identified to the lowest taxonomic category, at least to the family level. The density of benthic organisms were standardized by cover area of the sampling tools.

Five kinds of fish collection methods were utilized in our study. Fishing in the downstream area was on boat during the flood tide period where the water depth was more than 1 meter, and both gill nets and cast-nets were used at the same time. Gill nets were composed of three layers of different bar size, their bar on both sides were 25 cm and 7.5 cm in the middle. The bar size of the cast net was 2.5 cm, and the circular rim length was 4.5 meters. At the river mouth close to the sea, shrimp traps (collapsible shrimp traps, also called snake cage) used by local fisherman were also set overnight to catch the benthic fishes. The shrimp trap could catch the fish which were not easily caught by the gill net and cast-net. Electrofishing and shrimp traps were used in the upstream area to collect fish living on the rugged river bed. Electrofishing was conducted for 20 min, searching on 50 m along the river bed against the current in a zigzag track. The electrical power source was 12 V direct current (DC), and the discharge current was 110–150 V. The effected discharge field width of electrofishing was 1.5–2 m. The cast-net method was used on upstream riverbanks to catch fish living in deep pools and running water; the net size was the same as in the downstream area. Each upstream sampling site set six shrimp traps with pig liver and fermented soy bean cake as bait to attract fishes and shrimps. The shrimp traps were immersed in shallow water for 24 h before retrying. The fishes collected by different methods were identified to the species level. For some specimens we had to use genetic identification by the barcoding method.

The fishing effort was standardized in two ways. First, we sampled each station using the same effort (same duration of working period, same fishing gear and sampling area, etc.) each season. Secondly, we used a sampling area (cover area by net) and time consumption to be an indication to standardize the effort between each station. The electrofishing fishing efforts were standardized by total area of the electric discharge-affected field. The shrimp trap fishing effort was considered a single unit, and catch content only represented the species richness.

### 2.3. Community Structure Analysis and Biological Indexation

We employed canonical correlation analysis to describe the relationships between aquatic environmental factors and the structures of the biological community. We used two different strategies to analyze the community structure of benthic invertebrates and fish, and their relationships with aquatic environmental factors. The mobility of benthic invertebrates is low, so sampling sites revealed the pollution effects on location. We treated benthic invertebrate assemblages of each site as an independent data point, with all data points pooled together in analysis. The fish were mobile in the water mass, so water quality at a sampling site was more important than sampling location. The fish community analysis was based on the difference of water mass, upstream pure freshwater pooled as a unit and downstream area affected by tidal movement pooled as another unit. These biological taxa were included in canonical correlation analysis based on their abundance and distribution. Some taxa were eliminated in this analysis, when the taxon was only collected once and had less than two individuals. For each taxon, the abundance (*x*) was log normalized by log (*x*+1) to reduce the variations. The analysis was performed using the CANOCO 4.5 statistical program [31].

For each sampling site, we use the Shannon-Weaver index information statistic to measure the diversity. The Shannon diversity index for real communities often falls between 1.5 and 3.5, with the higher the value, the greater the diversity. For the benthic invertebrate community, the Hilsenhoff Family biotic Index (FBI) [28] was derived based on the pollution tolerance value of each taxon. A higher FBI score indicated that the water quality was worse. The tolerance value of each taxon was modified from many authors’ publications [32,33,34,35,36], and adjusted according to the canonical correlation analysis results (details are provided in Table A2). The Biological Index (BI) of benthic invertebrate animals [9] was derived from the saprobic level of each taxon. A higher BI score indicated that the water quality was better. For the fish community, Index of Biotic Integrity (IBI) was derived from standardized catch data, and 10 ecological character items modified from Karr [30,37,38] were employed in our study. In our calculation, each item scored from 1 to 5 points, so the total score was 50 points to indicate the best water quality. The catch weight and percentage of abnormal individuals suggested by Karr was not evaluated in our IBI index. The catch weight could be very variable in our study, as some big fish might be thousands of times heavier than small fish. Abnormal fish were rare in this study based on our standards, and not easy to identify objectively in the wild. The pollution tolerance value of fishes was modified from the EPA Taiwan handbook and other author’s reports [39,40,41,42,43], and adjusted according to the results of canonical correlation analysis. The formulation and correlated categorization of water quality for FBI, BI and IBI is listed in Table A3.

## 3. Results and Discussion

### 3.1. River Pollution Index

**Figure 2:**
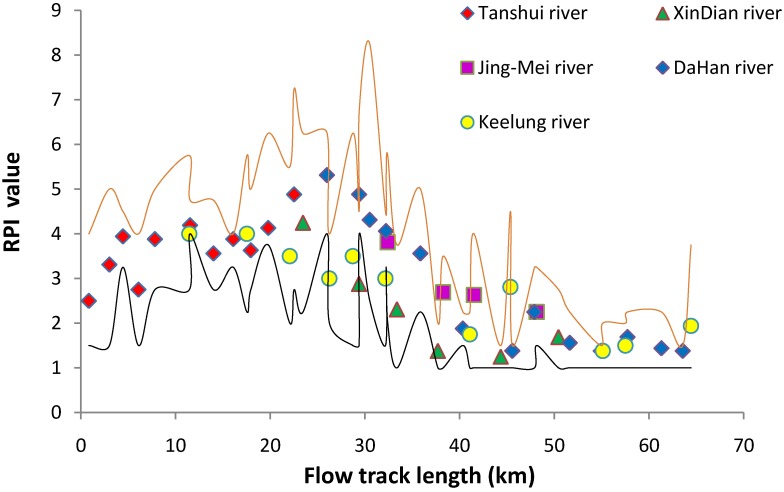
The RPI value of the Tanshui River system in 2012. The point marks indicate the mean of four seasons, red line was upper limit and black line was lower limit. RPI under 2 Non-impaired, RPI 2.0–3.0 Slightly impaired, RPI 3.1–6.0 moderately impaired, and RPI above 6 means severely impaired.

According to the river pollution index (RPI) of each sampling site in 2012, the most seriously impaired waters at flood tide period were located in a range of 10 to 35 km area relative to the river mouth as zero point (Figure 2). Based on the RPI ranking, this area corresponded to moderately impaired water most of the time and in some cases with severe pollution when the flow volume was small. In the lower part less than 10 km form the river mouth the pollution level decreased gradually and became negligible because of the massive sea water dilution effect. In the upper part of the river basin more than 35 km away, the pollution decreased gradually as it was more inside the mountain ranges with low human population. Upstream areas at distances greater than 50 km was lightly polluted or unimpaired most of the time. The 35 km flow track is also the upper limit of the tidal effect of the estuary, and most of the downstream retained water was moved back and forth between the 15–35 km range in a single tidal cycle. The polluted water could not be sent to sea by only a single tidal flow, and retained water in the fixed region between the 15–35 km range became more polluted by accumulating more pollutants. During the ebb tide, the most polluted waters would move downward to the sea. The volume of cleaning freshwater from upstream was the key factor determining the pollution level of the tide-affected area at ebb tide.

### 3.2. Species Richness and Distribution Pattern

From all the collections of 45 sampling sites in four surveys, 98 taxa of macroinvertebrates and 88 species of fishes were collected. The macroinvertebrates included 17 mollusk taxa, one ribbon worm (Nemertea) taxon, 10 annelid taxa, 24 crustacean taxa and 46 aquatic insect taxa. The downstream tide-affected region was dominated by mollusks and annelids without aquatic insects, except for some blood worms (larvae of nonbiting midges) we could find at the top of this region near the freshwater basin (X3, D4 and K6). The upstream freshwater water basin was dominated by diversified aquatic insects. The lowest diversity of benthic macroinvertebrate community among all sampling sites was found between the 15–35 km flow track length range, related to RPI distribution. In this region we could only find pollution tolerant species, and the Shannon-Weaver diversity index was mostly less than 0.5.

Biological surveys of the Tanshui River and its related branches began after 1970, most studies focusing on lower part of the Keelung River and Tanshui River. The key investigation item was the fish ensemble and benthic invertebrates. According to the organized archives, survey results from 1980 to 2005 [44], the fish species ensemble and benthic invertebrates of the lower region were severely affected by water pollution after 1980. According to the study of Houng and Hu [4], 11 species of aquatic insects belonging to six orders could be found at X2 of Xindian River in 1974. In this study, sites X2 and X3 only plenty of blood worms could be found. The substrate of X2 and X3 is sandy or muddy without rocks and pebbles at present, which also differs from earlier times. The modification of the river bed that altered the substrate might also play some role on changing the distribution of aquatic insects.

In the past decades, sewage pollution input large amounts of detritus and nutrients into the Tanshui River. Primary production may increase as nutrients subsides in the estuary ecosystem without light limitation. According the study by Lin *et al*. [45], the trophic structure of the polluted Tanshui River ecosystem was mostly supported by the detritus food chain not by primary production. Fish and invertebrate bottom feeders dominating the system feed on detritus particles on the river bed, with a lack of surface filter feeders and piscivorous fish. Primary production was limited by murky waters, which was caused by the abundant detritus particles and suspended solids (SS). Limited photosynthesis let unassimilated nutrients transported by river flow reach the coastal waters of the Taiwan Strait.

In 2012, the composition of the fish community could be roughly separated into two different spatial types, the estuarine community with 44 species and the freshwater community with 48 species. Some species that could migrate into the lower part of the freshwater basin and were included in the upstream freshwater community were *Mugil cephalus*, *Oreochromis* spp., *Chanos chanos* and *Megalops cyprinoides*. From river mouth to the lower part of the freshwater basin, the most common fish species were *Mugil cephalus* and *Oreochromis* spp., both of them with huge populations. Similar to the case of the invertebrate community, the lowest fish community diversity was also found between the 15–35 km range, with a Shannon-Weaver diversity index of less than 1 relative to other regions with an diversity index greater than 1. The Tanshui River had an important freshwater fishery and shipping industry before 1945 under Japanese rule. The most famous fishery production in the past was native sweet smelt (*Plecoglossus altivelis*) [46]. The hatchery ground of sweet smelt was at the historical Liu Gong irrigation canal connected to the Xindian River (now near Taiwan University at X2). After hatching, the larval sweet smelt were transported downstream to the Tanshui River mouth, and young fish would migrate upstream to the freshwater region to grow up. The sweet smelt population in Tanshui River decreased rapidly in the early 1900s. The major reason was overfishing, and they became extinct in the 1970s when water pollution cut off their migration route [47]. The Tanshui River canal silted up rapidly after 1945, and shipping and fishery activities were limited to a narrow region from Twa-tiu-tiann (site T10) to the river mouth. Invasive cichlid fishes (*Oreochromis* spp. and *Tilapia* spp.) dominated the downstream area of the river on the west side of Taiwan including the Tanshui River in 1970s [3]. Cichlids had a great impact on the native fish species and altered the fish community structure in many ways [47].

The fish species richness of the Tanshui River reached its lowest point in the 1990s. The fish species richness has increased significantly recently, and the species record of the entire sampling region was more than 100 including the records in 2009 [48]. The freshwater fish species list of freshwater basin was similar to that of many decades ago, although some invasive species have become very common. The increase of species richness happened mostly in the estuarine region, the flow track from the river mouth to the less than 35 km area. Relative the historical records, the species richness of the estuary between 1998 and 2005 had no more than 20 species, 43 recorded species in 2009 [48] and we identified 44 species in this study.

The increase of fish species richness indicated more estuarine fish got adapted to pollution mitigated waters. During the flood tide period in 2012, planktivorous species and piscivorous species migrated into the river reaching sites T10 and K3 sometimes. The most common planktivorous fishes were Clupeidae, Engraulidae, Leiognathidae and other juvenile fish. The piscivorous fish include *Lutijanus* spp., *Caranx* spp. and *Lateolabrax japonicas*. The trophic level structure was more diversify than in 2007 as described by Lin *et al*. [45]. During the rainy season with low pollution levels, some planktonic larvae of catadromous fish or crustaceans also could cut across this region into the upstream freshwaters. The most important species was fat snout rock climbing gobies (*Sicyopterus japonicas*) and Japanese Mitten crab (*Eriocheir japonicas*), which were very common before the water was polluted. Recent records of fat snout rock climbing gobies were at the Xindian River (Byitan Bridge Station: Figure 1, sampling site X4) in 2008 [48], and it was also found in this study at the same site. Before 2008, around the Tanshui River basin there were no records of fat snout rock climbing gobies for at least for 40 years. A similar situation occurred with the Japanese Mitten crab, the latest record was this study, at the collection site on the Keelung River (Changan bridge station: Figure 1, sampling site K7). Some other catadromous river shrimp *Macrobrachium formosense*, *Macrobrachium jaopnicum* and *Macrobrachium austral* were more easily found in the upstream freshwater, which was dominated by the land lock species *Macrobrachium aspcrulum*.

### 3.3. Community Structure and Environmental Factor Correlations

**Figure 3:**
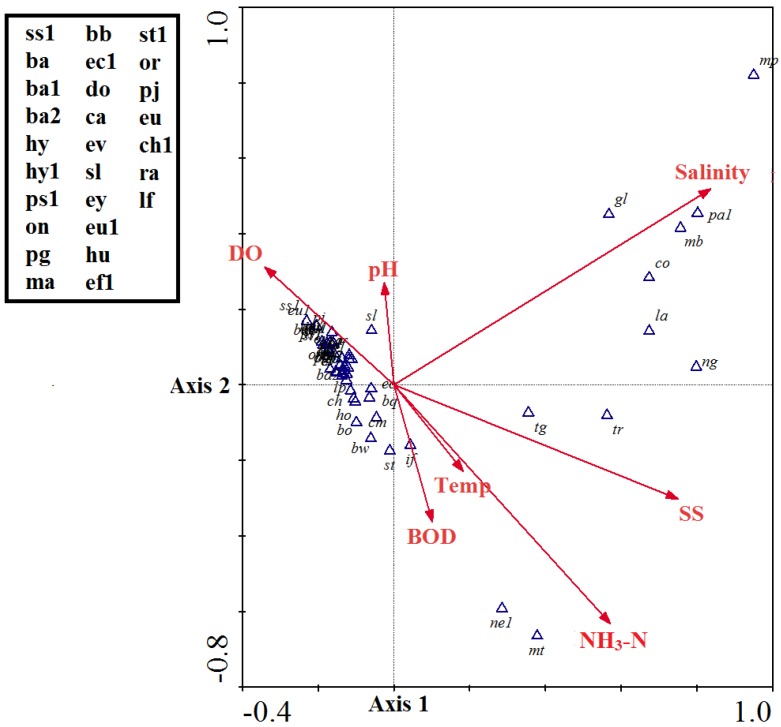
The environmental factors loading and their correlation to macro-invertebrates taxa of the Tanshui River system in 2012. Plot based on the regression on factor 1 and factor 2. The abbreviations used in this picture are listed in Table A2. The abbreviations overlapped in dense groupings in the center of figure are relist in the square box.

Seriously impaired water with low oxygen content and high ammonia nitrogen is a poor habitat for aquatic animals. The sedentary benthic animals could not accommodate the pollution waters in the short term and have no way of avoidance, so the succession of the community mostly depends on the pollution levels. The community structure of benthic macroinvertebrates was well correlated to environmental factors in this study. The taxa-environment factor correlations (*r*) for factor 1 and factor 2 were 0.88 and 0.68, respectively, even the taxa abundance between each sampling with large variation (Table 1). The distribution of taxa and factors loading on the plan of factors 1 and 2 is illustrated in Figure 3. Most aquatic insects were distributed at the low salinity water which had higher DO and lower SS, BOD_5_ and ammonia nitrogen content. In the estuary with saline water could find limited taxa which were tolerant of pollution. The effects of key pollution factors SS, BOD_5_ and ammonia nitrogen were similar way and correlated with the distribution of limited high pollution tolerant taxa. The temperature indication also fit to the group with higher temperature in the downstream polluted area with low DO, and the cumulative percentage variance of species-environment relation for first and second factor combinations was 64%.

The distribution of aquatic insects depended on substrate types and DO in waters. The substrate of upstream river bed was rugged, with rocks and pebbles, and the aeration by many small falls on rocks increases the DO in water. Impaired water of the downstream area with high content of BOD_5_ and ammonia nitrogen kept the DO at low levels by consuming oxygen continuously. The sandy or muddy substrates under deep water lack aeration effects unlike the upstream areas, and there was not enough oxygen for the survival of aquatic insects.

**Table 1:** The result of canonical correlation analysis between environmental factors and benthic macro-invertebrate taxa of each sampling sites of the Tanshui River system in 2012.

Axes	1	2	3	4	Total inertia
Eigenvalues	0.719	0.367	0.212	0.182	10.375
Taxa-environment correlations (*r*)	0.887	0.682	0.739	0.592	--
Cumulative percentage variance	--	--	--	--	--
Taxa	6.9%	10.5%	12.5%	14.3%	--
Taxa-environment relation	42.5%	64.2%	76.8%	87.5%	--
Sum of all eigenvalues	--	--	--	--	10.375
Sum of all canonical eigenvalues	--	--	--	--	1.69

**Figure 4:**
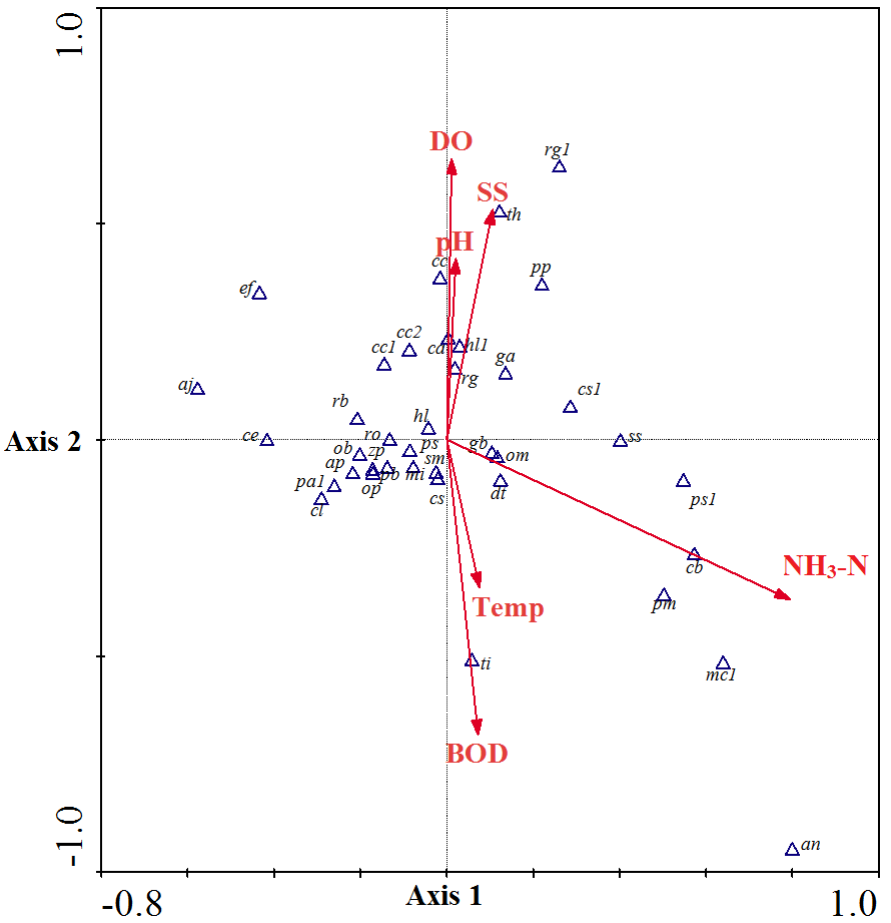
The environmental factors loading and their correlation to fish taxa of the Tanshui River system. Sampling sites located in upstream area were not affected by tidal movement in 2012. Plot based on the regression on factors 1 and 2. The abbreviations used in this picture are listed in Table A2.

Fish move continuously to locate the best aquatic environment for surviving. In the downstream estuary community, the water salinity was affected by tidal mixing on different scales. The distribution of marine species in this area was restricted by salinity as the first factor. The upstream area without tidal effects was designated as a freshwater community without large scale salinity variations. In the upstream freshwater fish community, taxa abundance showed large variations between each sampling. The taxa-environment factor correlations (*r*) for factors 1 and 2 were 0.66 and 0.63, respectively, and the cumulative percentage variance of species-environment relation for first and second factor combinations was 65% (Table 2). The distribution of taxa and factors loading on the plan of factor 1 and factor 2 is shown in Figure 4. The effect of the pollution factor ammonia nitrogen was more independent than other factors, and combined with the effect of BOD_5_ could group pollution tolerance of taxa living in the waters with lower DO. The other group was pollution intolerant taxa who lived in waters with higher DO and lower concentration of ammonia nitrogen and BOD_5_.

**Table 2:** The result of canonical correlation analysis between environmental factors and fish taxa of the Tanshui River system in 2012. Sampling sites were divided into two groups, upstream area without tidal effect and downstream area with tidal effect.

Axes	1	2	3	4	Total Inertia
Upstream area without tidal effect					
Eigenvalues	0.139	0.115	0.057	0.039	3.33
Species-environment correlations	0.661	0.63	0.622	0.578	--
Cumulative percentage variance	--	--	--	--	--
Species	4.2%	7.6%	9.3%	10.5%	--
Species-environment relation	35.5%	65%	79.6%	89.5%	--
Sum of all eigenvalues	--	--	--	--	3.33
Sum of all canonical eigenvalues	--	--	--	--	0.391
Downstream area with tidal effect					
Eigenvalues	0.505	0.208	0.098	0.087	5.656
Species-environment correlations	0.869	0.792	0.644	0.602	--
Cumulative percentage variance	--	--	--	--	--
Species	8.9%	12.6%	14.3%	15.9%	--
Species-environment relation	50.3%	71%	80.7%	89.4%	--
Sum of all eigenvalues	--	--	--	--	5.656
Sum of all canonical eigenvalues	--	--	--	--	1.004

**Figure 5:**
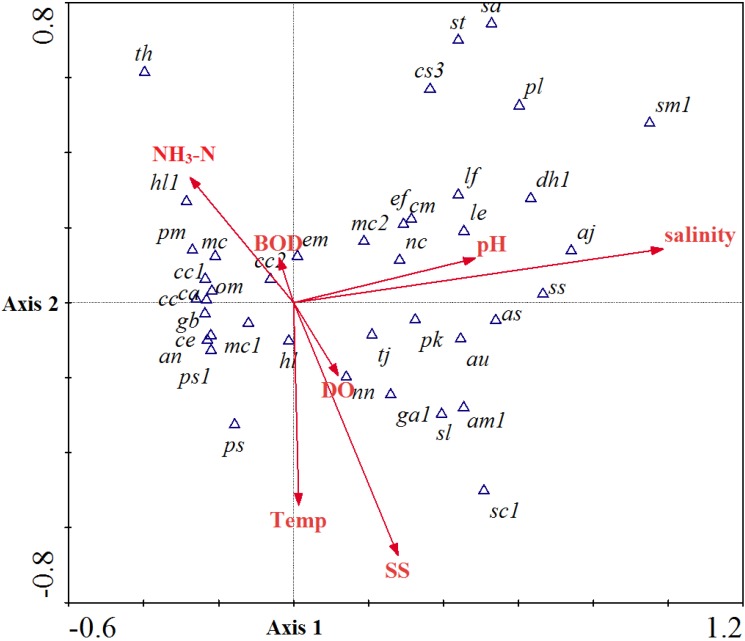
The environmental factors loading and their correlation to fish taxa of the Tanshui River system. Sampling sites were located in the downstream area and were affected by tidal movement in 2012. Plot based on the regression on factors 1 and 2. The abbreviations used in this picture are listed in Table A2.

The taxa abundance showed large variations between each sampling in the downstream area in the estuary. The taxa-environment factor correlations (*r*) for factor 1 and factor 2 were 0.86 and 0.79, respectively, and the cumulative percentage variance of species-environment relation for first and second factor combinations was 71% (Table 2). The distribution of fish taxa mostly depended on tidal movement and salinity (Figure 5). The effects of the pollution factors ammonia nitrogen content was stronger than BOD_5_, but it affected the community structure in a similar way. The groups of tolerant taxa could live in the waters with lower salinity, that indicates these fishes could migrate upstream and cross over seriously impaired waters into the freshwater region. Some fishes stayed in the upstream area and did not go out during ebb tide. The size of the effect of DO and BOD_5_ on the downstream community was much smaller than in the upstream freshwater area. The flood tide dilution effects might be the major reason, as fishes moved up and down depending on the water quality and their individual tolerance.

The effect size of SS was only smaller than salinity and was in second place in the Tanshui River. Suspended solids can clog fish gills, either killing them or reducing their growth rate. Suspended solids also reduce light penetration. This reduces the ability of algae to produce food and oxygen. Most suspended solids in the Tanshui River were from anthropogenic sources with large scale increases being caused by heavy rains during the typhoon season. When the water slows down downstream, the silting of suspended sediments changes the river bed to muddy and flat. Truncation curved cut-off and canalization that altered the river physically might also affect the siltation process and location. According to Appleby and Scarratt [49], estuary fish and shellfish survival in concentrations of SS under experimental conditions was varied and the limitations greater than those commonly observed in Nature. The SS concentration in the upstream area of the Tanshui River system was less than 25 (mg/L) usually, thus corresponding to unharmful conditions as suggested by Ward in 1992 [50]. Little siltation occurred in the upstream area, where the rapid flow very easily carried away SS.

### 3.4. Biological Indexation

**Figure 6:**
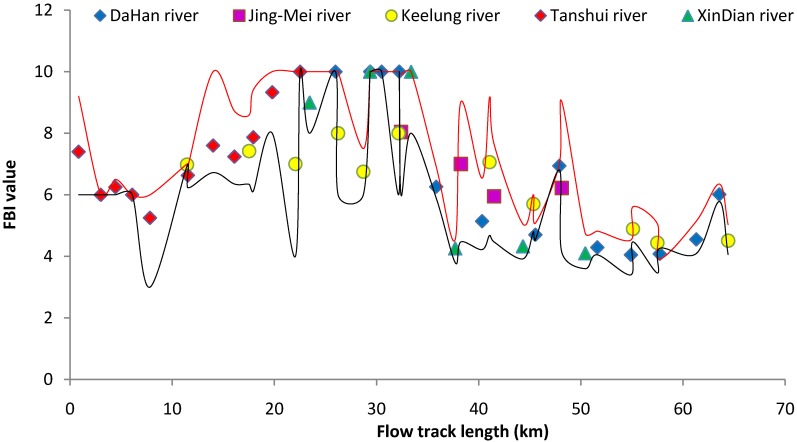
The FBI value for the benthic macroinvertebrate community of the Tanshui River basin in 2012. The point marks indicate the mean of four seasons, red line was upper limit and black line was lower limit.

**Figure 7:**
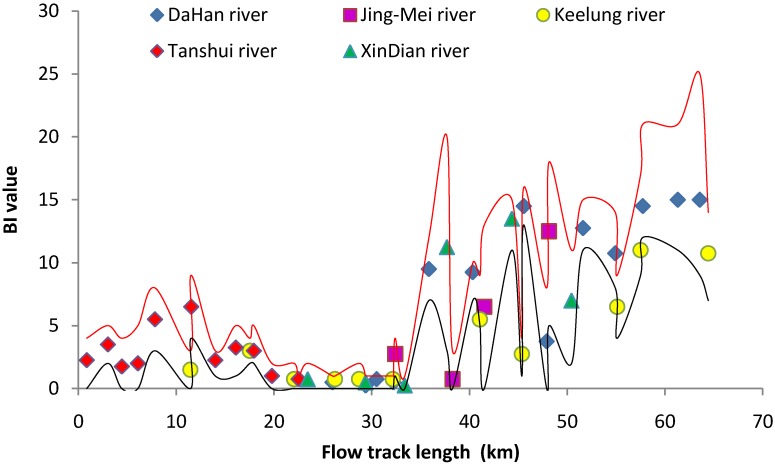
The BI value for the benthic macroinvertebrate community of the Tanshui River basin in 2012. The point marks indicate the mean of four seasons, red line was upper limit and black line was lower limit.

Hissenhoff Family Biotic Index (FBI) and the Suisei Konchūgaku Biotic Index (BI) both indicated the same conditions for the benthic environment of the Tanshui River (Figure 6 and Figure 7). The benthic substrate environment of the tide-affected area (0–35 km flow track length) was still unfavorable for macroinvertebrates. The water quality of the Tanshui River system including the major branches had been assessed in 1979 by BI derived from benthic invertebrate assemblage [5]. The benthic substrate environment of the tide-affected area in 2012 was similar to that in 1979, and species richness and diversity was low at most of the sampling sites. The unfavorable benthic substrate conditions persist many years, and only limited high pollution tolerant species could colonize here. Even after pollution mitigation, the benthic substrates are still under anoxia conditions with high concentrations of organic carbon and other toxic materials [51]. Benthic invertebrates find it more difficult to recolonize than fish and other organisms living in waters that respond to water quality improvements. According to rapid the biological assessment charts study by Besley and Chessman [8], benthic invertebrates recovered very slowly after the pollution source was eased 7 years ago in small streams in the Blue Mountains, Australia.

Relative to the estuarine region, the substrate of the freshwater basin as indicated by the FBI and BI represent that the environment was more favorable for benthic invertebrates (Figure 6 and Figure 7). Aquatic insects were the key components of the benthic invertebrate community. The stone fly (Plecoptera) indicated that a well oxygenated habitat with running water and low temperature habitat could only be found at most upstream sampling sites. Mayfly (Ephemeroptera) and caddies fly (Trichoptera) were widely distributed in the freshwater basin, which could be a good indication of clean water. The aquatic insects also were key food items of fishes in the freshwater basin.

**Figure 8:**
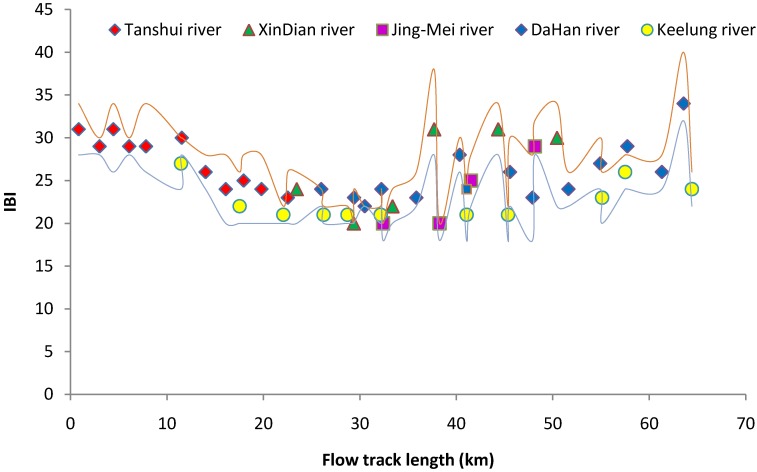
The IBI value for the fish community of the Tanshui River basin in 2012. The point marks indicate the mean of four seasons, red line was upper limit and black line was lower limit.

The Index of Biotic Integrity (IBI) for the fish community indicated another scenario of pollution conditions. The distribution of IBI (Figure 8) was closely related to the RPI distribution (Figure 2). Between the regions of track length 15 km to 35 km, the water quality was most unfavorable for fish in the Tanshui River. During flood tides, most low pollution tolerant estuarine species follow the rising sea water moving deep into the river, and reach the 15 Km track length. The higher pollution tolerant estuarine species could reach to the upper limit of tide-affected area (35 track length), and some species even moved into the freshwater in the short term.

During the ebb tide, the entire lowland area region (track length less than 35 km) lacked the dilution by sea waters effect. When clean freshwater input from upstream was rare, the lowland area was a poor habitat for fish. The predicted RPI change by tidal movement in the Tanshui River by kriging analysis [52] also can be a good indication of fish community variation at a specific time and location of river. Above the 35 km track length, the variation of IBI was more significant than in the lower region. The variation might depend on the natural conditions of the different sampling sites. The most important effect was flow volume and flow rate of the river, which were determined by precipitation distribution. During the typhoon season, the large volume of freshwater could destroy the aquatic habitat entirely in the short term, also could create new habitats and decrease the concentration of anthropogenic pollutants of the entire water body by the dilution effect.

Water quality indicated by fish assemblage was much better than for benthic invertebrates in this study. The results of canonical correlation analysis revealed that the assemblages of the two groups were influenced by different sets of environmental drivers. Weak concordance between fish and macroinvertebrates was also presented in other studies [53,54,55,56]. The improvement of water quality did not result in an improvement of substrate habitat for benthic animals. The monitoring programs for the Tanshui River should still consider a multi-assemblage assessment rather than a single group of organisms. The same requirement is also required by the Water Framework Directive (WFD) of the European Union, using multimetric indices and multivariate statistical methods to uncover patterns in the taxonomical composition-related environmental variables [26].

## 4. Conclusions

In Taipei City, like many other big cities in the world, urbanization, industrialization, and human population growth are causing serious environmental problems. The Tanshui River is the major river flowing through the city and has been severely polluted and its aquatic habitats destroyed in many ways. The rehabilitation of the Tanshui River after pollution levels decreased in recent years was a great achievement. Multivariate statistical analysis has uncovered the patterns in the taxonomical composition of invertebrates and fish related to the levels of water pollution. The effects of key pollution factors SS, BOD_5_ and ammonia nitrogen were similar way and correlated with limited high pollution-tolerant taxa. Species tolerance values cited from references were adjusted according to the results of canonical correlation analysis.

Multimetric indices were derived from the benthic invertebrate and fish communities to represent the ecological status of the Tanshui River. The integrity of the fish community indicated by the IBI increased as water quality improved in all the Greater Taipei basin. Some planktonic larval stages of catadromous fish and crustaceans also could across the moderately polluted water into the freshwater region upstream and re-colonize their populations. The water quality improvement was achieving the same result for the benthic macroinvertebrate communities that rely on substrates. The benthic habitat indicated by FBI and BI is still very poor on the area with moderately polluted waters. Limited species could endure anoxic substrates with high concentrations of organic carbon and toxic substances. More work has to be done on substrate sludge cleaning or to speed up active decomposition, increasing the diversity and integrity of the benthic community.

## Appendix

**Table A1:** River pollution index (RPI) using by EPA Taiwan, RPI under 2 Non-impaired, RPI 2.0-3.0 Slightly impaired, RPI 3.1-6.0 moderately impaired, and RPI above 6 belong to severely impaired.

Item	Non-impaired	Slightly impaired	Moderately impaired	Severely impaired
(DO) mg/L	D ≥ 6.5	6.5 < D ≥ 4.6	4.5 ≥ D ≥ 2.0	D < 2.0
(BOD_5_) mg/L	B ≤ 3.0	3.0 < B ≤ 4.9	5.0 ≤ B ≤ 15.0	B > 15.0
(SS) mg/L	S ≤ 20.0	20.0 < S ≤ 49.9	50.0 ≤ S ≤ 100	S > 100
(NH_3_-N) mg/L	N ≤ 0.50	0.50 < N ≤ 0.99	1.00 ≤ N ≤ 3.00	N > 3.00
Score	1	3	6	10
RPI= (D + B + S + N)/4	S ≤ 2.0	2.0 < S ≤ 3.0	3.1 ≤ S ≤ 6.0	S > 6.0

**Table A2:** Fish taxa and macroinvertebrate taxa, code for Figure 3, Figure 4 and Figure 5 and pollution tolerance values used in biological indication for Figure 6, Figure 7 and Figure 8 are listed.

Taxa	Code	Tolerance value
Fish		
**Anguillidae**		
*Anguilla japonica*	aj	4
*Anguilla marmorata*	am	4
Cyprinidae		
*Rhodeus ocellatus*	ro	5
*Tanakia himantegus*	th	5
*Microphysogobio brevirostris*	mi	3
*Acrossochelius paradoxus*	ap	3
*Aristichthys nobilis*	an	5
*Candidia barbata*	cb	2
*Carassius auratus*	ca	6
*Carassius cuvieri*	cc	6
*Ctenopharyngodon idella*	ci	6
*Chanodichthys erythropterus*	ce	5
*Cyprinus carpio carpio*	cc1	6
*Distoechodon tumirostris*	dt	5
*Hemibarbus labeo*	Hl	6
*Hemiculter leucisculus*	hl1	6
*Pseudorasbora parva*	pp	5
*Sinibrama macrops*	sm	4
*Onychostoma barbatulum*	ob	0
*Opsariichthys pachycephalus*	op	3
*Zacco platypus*	zp	3
*Mylopharyngodon piceus*	mp	5
*Crossostoma lacustre*	cl	2
*Hemimyzon formosanum*	hf	1
**Cobitididae**		
*Cobitis sinensis*	cs	2
*Misgurnus anguillicaudatus*	ma	6
**Bagridae**		
*Pseudobagrus adiposalis*	pa	2
*Pseudobagrus brevianalis*	pb	2
**Siluridae**		
*Silurus asotus*	sa	6
*Pangasius sutchi*	ps	8
Siganidae		
*Siganus canaliculatus*	sc	3
**Loricariidae**		
*Pterygoplichthys multiradiatus*	pm	9
*Hemichromis bimaculatus*	hb	8
*Oreochromis mossambicus*	om	8
**Tilapia sp.**	ti	8
*Paratheraps synspilum*	ps1	8
*Labidochromis caeruleus*	lc	8
**Mugilidae**		
*Chelon macrolepis*	cm	8
*Mugil cephalus*	mc1	7
*Moolgarda cunnesius*	mc2	7
*Chelon subviridis*	cs3	7
Synbranchidae		
*Monopterus albus*	ma1	5
**Eleotridae**		
*Eleotris fusca*	ef	3
**Gobiidae**		
*Sicyopterus japonicus*	sj	3
*Rhinogobius brunneus*	rb	3
*Rhinogobius giurinus*	rg	4
*Rhinogobius gigas*	rg1	3
**Moronidae**		
*Lateolabrax japonicus*	lj	3
**Channidae**		
*Channa striata*	cs1	9
**Diodontidae**		
*Diodon holocanthus*	dh1	3
**Sillaginidae**		
*Sillago sihama*	ss	3
**Sphyracenidae**		
*Sphyraena putnamae*	sp	3
**Lutjanidae**		
*Lutjanus fulviflamma*	lf	3
**Apogonidae**		
*Apogonidae* sp.	ap1	3
**Haemulidae**		
*Pomadasys kaakan*	pk	3
**Serranidae**		
*Epinephelus coioides*	ep	3
*Epinephelus radiatus*	er	3
**Elopidae**				
*Elops machnata*	em	9		
**Megalopidae**				
*Megalops cyprinoides*	mc	9		
**Leiognathidae**				
*Nuchequula nuchalis*	nn	3		
*Nuchequula mannusella*	nm	3		
*Leiognathus equulus*	le	3		
**Ambassidae**				
*Ambassis urotaenia*	au	3		
**Gerreidae**				
*Gerres abbreviatus*	ga1	3		
**Scatophagidae**				
*Scatophagus argus*	sa	9		
**Sparidae**				
*Rhabdosargus sarba*	rs	3		
*Acanthopagrus latus*	al	3		
*Acanthopagrus schlegelii*	as	3		
**Ophichthidae**				
*Pisodonophis cancrivorus*	pc	3		
**Sciaenidae**				
*Johnius dussumieri*	jd	3		
**Clupeidae**				
*Sardinella melanura*	sm1	3		
*Sardinella lemuru*	sl	3		
*Sardinella albella*	sa1	3		
*Nematalosa come*	nc	3		
**Engraulidae**		
*Thryssa hamiltonii*	th1	3
*Thryssa chefuensis*	tc	3
**Chanidae**		
*Chanos chanos*	cc2	5
**Carangidae**		
*Scomberoides tol*	st	3
*Scomberoides commersonnianus*	sc1	3
*Caranx sexfasciatus*	cs2	3
*Carangoides malabaricus*	cm1	3
**Macroinvertebrates**		
**Mollusca**		
**Thiaridae**		
*Stenomelania torulosa*	st	3
*Melanoides tubercufatus*	mt	3
*Tarebia granifera*	tg	3
*Thiara riqueti*	tr	3
**Pleuroceridae**		
*Semisulcospira libertina*	sl	3
**Viviparidae**		
*Bellamya quadrata*	bq	6
**Corbiculidae**		
*Corbicula fluminea*	cf	4
**Mytilidae**		
*Limnoperna fortunei*	lf	6
*Perna viridis*	pv	6
**Veneridae**		
*Meretrix petechialis*	mp	
**Philinidae**		3
*Physa acuta*	pa	8
**Lymnaeidae**		
*Lymnaea pervia*	lp	6
*Radix auricularia*	ra	6
**Planorbidae**		
*Hippeutis umbilicalis*	hu	7
**Tellinidae**		
*Tellina virgata*	tv	3
**Unionidae**		
*Unio douglasiae*	ud	5
**Ampullariidae**		
*Pomacea canaliculata*	pc	8
**Nemertina**	ne2	10
**Annelida**		
**Polychaeta**		
**Phyllodocimorpha**		
**Goniadidae**		
*Glycinde* sp.	gl	6
**Nereididae**		
*Neanthes* sp.	ne	6
*Neanthes glandicincta*	ng	6
*Perinereis aibuhitensis*	pa1	6
**Sabellida**		
**Sabellidae**		
*Laonome albicingillum*	la	6
**Hesionidae**		
*Micropodarke* sp.	mi	6
**Hirudinea**		
**Rhynchobdellida**		
**Glossiophonidae**		
*Helobdella europaea*	he	9
*Alboglossiphonia lata*	al	9
*Barbronia weberi*	bw	9
**Erpobdellidae**		
*Bobronia* sp.	bo	9
**Arthropoda**		
**Malacostraca**		
**Amphipoda**		
**Melitidae**		
*Melitidae* sp.	me	6
**Corophiidae**		
*Corophium major*	cm	6
*Corophium* sp.	co	6
*Gammaropsis* sp.	ga	6
**Decapoda**		
**Palaemonidae**		
*Macrobrachium asperulum*	ma	6
*Macrobrachium nipponense*	mn	6
*Macrobrachium formosense*	mf	6
*Macrobrachium jaopnicum*	mj	6
*Macrobrachium australe*	ma1	6
**Atyidae**		
*Neocaridina denticulata*	nd	6
*Caridina pseudodenticulata*	cp	6
**Penaeidae**		
*Metapenaeus monoceros*	mm	6
*Penaeus* sp.1	mm1	6
**Cambaridae**		
*Procambarus clarkii*	pc1	6
**Potamidae**		
*Geothelphusa candidiensis*	ga1	6
**Ocypodidae**		
*Ilyoplax fromosensis*	if	6
*Uca borealis*	ub	6
*Macrophthalmus banzai*	mb	6
**Grapsidae**		
*Eriocheir japonicus*	ej	6
*Helice wuana*	hw	6
**Portunidae**		
*Scylla serrata*	ss	6
*Portunus sanguinolentus*	ps	6
**Isopoda**	is	8
**Potamidae**		
**Insecta**		
**Ephemeroptera**		
**Baetidae**		
*Baetiella bispinosa*	bb	5
*Baetiella joponica*	bj	5
*Baetiella* sp.1	ba	5
*Baetiella* sp.2	ba1	5
*Baetis* sp.	ba2	5
**Caenidae**		
*Caenis bella*	cb	6
*Caenis* sp.	ca	6
**Ephemerellidae**		
*Ephemerella* sp.	ep	1
*Torleya japonixa*	tj	1
*Torleya glareosa*	tg1	1
*Uracanthella* sp.	ur	1
**Ephemeridae**		
*Ephemera formosana*	ef	4
**Heptageniidae**		
*Ecdyonurus* sp.	ec	4
*Ecdyonurus viridis*	ev	4
*Ecdyonurus yoshidae*	ey	4
**Leptophlebiidae**		
*Choroterpes* sp.	ch1	2
*Thraulis* sp.	th	2
*Paraleptophlebia* sp.	pa2	2
**Odonata**		
**Euphaeidae**		
*Euphaea formosa*	ef1	6
**Gomphidae**		
*Gomphus* sp.	go	1
*Lanthus fujiacus*	lf1	1
*Ictinogomphus* sp.	ic	1
*Onychogomphus* sp.	on	1
**Plecoptera**		
**Perlodidae**		
*Neoperla* sp.	ne1	1
**Trichoptera**		
**Hydropsychidae**		
*Hydropsyche orientalis*	ho	4
*Hydropsyche* sp.	hy	4
**Hydroptilidae**		
*Hydroptila* sp.	hy1	4
**Philopotamidae**		
*Dolophilodes* sp.	do	3
*Wormaldia* sp.	wo	3
**Stenopsychidae**		
*Stenopsyche schmidi*	ss1	4
*Stenopsyche* sp.	st1	4
**Psychomyiidae**		
*Psychomyiella* sp.	ps1	2
**Ecnomidae**		
*Ecnomus* sp.	ec1	3
**Lepidoptera**	le	5
**Coleoptera**		
**Elminae**		
*Ordobrevia* sp.	or	4
*Stenelmis* sp.	st2	4
**Psephenidae**		
*Eubrianax granicolis*	eg	4
*Eubrianax* sp.1	eu	4
*Eubrianax* sp.2	eu1	4
*Psephenoides* *japonicus*	pj	4
*Ellipes* sp.	el	4
**Sialidae**		
**Corydalidae**		
*Protohermes grandis*	pg	4
**Diptera**		
**Chironomidae**		
*Chironomus* sp.	ch	7
**Tipulidae**		
*Antocha* *saxicola*	as	3
*Tipula* sp.	ti	3

**Table A3:** The formulation of FBI, BI and IBI, indication of water quality.

Formulation	Score and combination	Indication of water quality
FBI = Σ( *ai* × *ni*)/*N*	*ai*: tolerance value of *i* family *ni*: individuals of *i* family *N*: individuals in total	0.00~3.75 Excellent 3.76~4.25 Very good 4.26~5.00 Good 5.01~5.75 Fair 5.76~6.50 Fairly poor 6.51~7.25 Poor 7.26~10.0 Very poor
BI = 2A + B + O	A: number of intolerant species B: number of tolerant species O: number of undefined species	≥20: oligosaprobic zone (os) 11~19: *β* mesosaprobic zone (*β*ms) 6~10: *α* mesosaprobic zone (*α*ms) 0~5: polysaprobic zone (ps)
IBI = A + B + … + J	A: percentage of native species, 1 (≤ 33 %); 3 (33–66%); 5 (≥ 66%) B: percentage of benthic species, 1 (≤ 33 %); 3 (33–66%); 5 (≥ 66%) C: percentage of pelagic species, 1 (≤ 33 %); 3 (33–66%); 5 (≥ 66%) D: percentage of untolerant species, 1 (≤ 5 %); 3 (5–15 %); 5 (≥ 15%) E: percentage of tolerant species, 1 (≥15 %) ; 3 (5–15 %); 5 (≤ 5%) F: percentage of omnivorous species, 1 (≥40 %); 3 (20–40 %); 5 (≤ 20%) G: percentage of insectivorous species, 1 (≤ 5 %) ; 3 (5–20 %); 5 (≥20%) H: percentage of piscivorous species, 1 (≤ 3 %) ; 3 (3–10%); 5 (≥10%) I: catch per unit effort (CPUE) 1(≤ 100); 3(100–250); 5(≥250) J: percentage of exotic species, 1 (≥10 %) ; 3 (1–10 %); 5 (≤ 1%)	45~50: Excellent 37~44: Good 28~36: Fair 16~27: Poor <16: Very poor *no fish
